# Clinical and Contrast-Enhanced Ultrasound Echography Outcomes in Psoriatic Arthritis Patients after One Year of Continuous Therapy with Anti-TNF Drugs

**DOI:** 10.1155/2014/932721

**Published:** 2014-02-06

**Authors:** Claudio Bonifati, Fulvia Elia, Dario Graceffa, Fabrizio Ceralli, Elisa Maiani, Carlo De Mutiis, Francesco M. Solivetti

**Affiliations:** ^1^Centre for the Study and Treatment of Psoriasis, Department of Clinical Dermatology, San Gallicano Dermatologic Institute (IRCCS), Via Elio Chianesi 53, 00144 Rome, Italy; ^2^Radiodiagnostic Service, San Gallicano Dermatologic Institute (IRCCS), 00144 Rome, Italy; ^3^Department of Rheumatology, San Camillo-Forlanini Hospital, 00151 Rome, Italy

## Abstract

*Background.* We wanted to verify retrospectively the proportion of patients with psoriatic arthritis who were in remission after 1 year of continuous therapy with either etanercept or adalimumab. Remission was defined as the absence of both clinical and contrast-enhanced ultrasound (CEUS) findings suggestive of joint inflammation. *Patients and Methods.* The data of twenty-five patients with psoriatic arthritis were available for the clinical and CEUS evaluations before and after 1 year of continuous therapy with etanercept or adalimumab. The count of swollen (ACR66), tender (ACR68), and active inflamed joints (AJC) was used to measure the severity of joint involvement. PASI was used to score the severity of psoriasis. HAQ, DLQI, VAS pain, and VAS itching were administered to each patient before starting therapy and every 3 months, up to 1 year. *Results.* Eight (32%) out of twenty-five patients were in remission after 1 year of therapy with etanercept or adalimumab. A significant reduction of all clinical variables analysed was seen during the course of therapy. *Conclusion.* Although a significant proportion of patients achieved remission of arthritis after 1 year of effective anti-TNF therapy, the majority of them continued to have either clinical or CEUS findings suggestive of persistence of joint inflammation.

## 1. Introduction

An inflammatory involvement of the axial and/or peripheral joints has been reported to occur in a percentage ranging from 6% to 39% of psoriatic patients [1]. More and more data clearly indicate that psoriatic arthritis (PsA) must be considered a serious disease with the risk of permanent joint deformity with disabling consequences [2].

In the majority of cases, skin manifestations precede by several years signs and symptoms of arthritis that in turn can have a long paucisymptomatic course [3]. Therefore dermatologists play a privileged role in suspecting PsA at an early stage and refer selected patients to a consultant rheumatologist to confirm the presence of inflammatory arthritis.

The diagnosis of PsA primarily relies on highlighting a musculoskeletal inflammatory condition (joint, spine, or entheseal) [3]. The articular disease can be classified as “psoriatic” if CASPAR criteria are satisfied [4].

As regards the diagnosis and followup of PsA, they have been improved in recent years thanks to the introduction of magnetic resonance imaging (MRI) and ultrasound (US). These imaging techniques have proven to be more sensitive than traditional X-rays, mainly in the diagnosis of early PsA [5, 6]. With regard to the sensitivity of US imaging, this can be further improved by the use of contrast enhancement (CEUS) as previously reported [7, 8].

At present, no data are available concerning CEUS to monitor the response of joint inflammation in PsA patients taking either modifying drugs (DMARDs) or antitumor necrosis factor drugs (anti-TNF).

In the present retrospective observational study, we aimed at verifying the proportion of patients with PsA who, after 1 year of continuous therapy with either etanercept or adalimumab, did not present either clinical or CEUS signs of active joint inflammation.

Moreover, we evaluated the modification of a series of clinical variables, exploring both skin and joint status, every 3 months up to 12 months of anti-TNF treatment.

## 2. Patients and Methods

Twenty-five patients with psoriatic arthritis, 13 males (52%) and 12 females, were analysed in the present study.

The cohort included patients who were taking either etanercept (15 subjects, 60%) or adalimumab (10 subjects) continuously for 1 year and in whom CEUS findings were available at the start and after 12 months of the therapy. The subjects reported here started treatment with the above cited anti-TNF drugs between January 2011 and February 2012.

The diagnosis of inflammatory arthritis was confirmed by consultant rheumatologists (DG or FC) and classified as PsA in agreement with CASPAR criteria [4].

Etanercept was administered at a dose of 50 mg weekly. Adalimumab was administered at a dose of 40 mg biweekly. Three patients, with more extensive skin disease in the adalimumab group, received an induction dose of 80 mg followed by an injection of 40 mg after 1 week and continued with 1 administration biweekly.

### 2.1. Clinical Measures

The data were recorded in an electronic database at the start of the study and at each visit every 3 months, up to 1 year.

The degree of articular involvement was estimated by the count of the number of swollen (ACR 66) and tender (ACR 68) [9] joints. The actively inflamed joint count (AJC), resulting from the sum of the swollen and tender joint count [10], was also used to monitor the response of arthritis to the anti-TNF agents administered.

The presence or absence of enthesitis was assessed at the level of the following sites: (i) bilateral Achilles tendon insertions, (ii) medial femoral condyles, and (iii) lateral epicondyles of the humerus.

The severity of psoriasis was measured by means of the psoriasis area severity index (PASI) [11].

The degree of impairment of functional status due to joint involvement was evaluated using the Health Assessment Questionnaire (HAQ) [12]. The impact of skin lesions on the quality of life was evaluated by means of Dermatology Life of Quality Index (DLQI) [13].

The answers to the HAQ and DLQI referred to the week before each visit to the clinic.

Visual analogue scales graded from 0 to 100 were used to measure the level of joint pain (VAS pain) and itch (VAS itch) felt by the patients in the week prior to each clinical evaluation.

### 2.2. Laboratory Testing

Blood was collected from all patients for routine analyses, which included (i) blood count; (ii) blood protein levels; (iii) erythrocyte sedimentation rate (ESR); (iv) C-reactive protein (CRP); (v) creatinine; (vi) uric acid; and (vii) urine analysis. The samples were collected before etanercept or adalimumab was started and 1–3 days before each scheduled clinical control performed every 3 months. Before the start of administration of the anti-TNF drugs, the following blood tests were performed on all patients: (i) quantiferon TB Gold; (ii) rheumatoid factor; and (iii) anti-nuclear antibody (ANA). All these tests were found to be negative.

### 2.3. Imaging Studies

After written consent, all patients were evaluated by ultrasound (US) before (basal ultrasound) and after (CEUS) intravenous bolus administration of US contrast agent (Sonovue Bracco, Milan, Italy), as previously reported [7, 8]. In particular, before starting the anti-TNF agents, one of the most active or clinically suspicious joints in each patient was selected for US and CEUS studies (Table 1). The same joints were examined after 1 year of anti-TNF treatment.

Intra-articular enhancement was graded following injection of the contrast agent using a 0–3 scale: grade 0: no intra-articular enhancement; grade 1: light enhancement; grade 2: moderate enhancement; and grade 3: severe enhancement [7, 8].

### 2.4. Remission Criteria

Patients were considered in remission when clinical signs of joint inflammation (tender and swollen joint count = 0, without signs of enthesitis or dactylitis) and CEUS results were negative at the 12th month of treatment.

### 2.5. Statistical Analysis

Nonparametric tests were used for comparisons given that data were not normally distributed. Specifically, Wilcoxon, Friedman, Kruskal-Wallis, and Mann-Whitney *U*-tests were used as necessary for ordinal data. McNemar and *χ* ^2^ tests for paired and unpaired data were used respectively, for nominal data. Statistical analyses were performed using Analyse-it software for Microsoft Excel, version 2.20 (Analyse-it software, Ltd. http://www.analyse-it.com/; 2009).

## 3. Results

The demographic characteristics of the patients are shown in Table 2. The median age of the cohort of subjects was 51 years (range 27–69 years). The duration of their psoriasis, median 23 years (range 2–47 years), was significantly longer than that of arthritis, median 3 years (range <1–31 years; *P* < 0.0001). In 7 (28%) out of 25 patients, their PsA was classified as “early” because its duration was ≤1 year [8].

Eighteen (72%) out of 25 patients with a duration of inflammatory arthritis ≤7 years received their first suspected diagnosis of PsA at our dermatology clinic which was confirmed by consultant rheumatologists (DG or FC). The remaining 12 subjects were diagnosed with PsA in clinical settings external to our clinic. As regards these latter patients, the presence of PsA was confirmed by consultant rheumatologists (DG or FC).

All but one patient (patient Number 9) received one or more courses with the following “traditional drugs” for either psoriasis or PsA: (i) acitretin; (ii) cyclosporine; (iii) methotrexate; (iv) prednisone; and (v) NSAIDs before starting the anti-TNF agents listed in Table 2.

Adalimumab or etanercept was administered to this cohort of the patients because the above-listed “traditional drugs” were of scarce efficacy in controlling their skin and/or articular symptoms or as a consequence of side effects. Moreover, before starting the anti-TNF drugs shown in Table 2, five subjects were previously treated with the following biological drugs: (i) etanercept (patient numbers 4, 14, and 15); infliximab (patient numbers 2, 4, 6, and 14); efalizumab (patient number 4); and golimumab (patient number 15). The previously administered biological therapies were interrupted due to primary or secondary failure, or for the appearance of severe adverse events. Out of 25 patients analysed herein, four (patient numbers 4, 6, 10, and 11) continued methotrexate therapy which they were taking prior to taking etanercept or adalimumab. In patient number 2 and number 26 methotrexate was added after starting TNF inhibitors due to a flare of arthritis and psoriasis, respectively.

The degree of joint involvement, presence or absence of dactylitis and enthesitis, and psoriasis area severity index scores (PASI) together with CEUS results, at the start and after the 12th month of the study, are summarized in Table 2.

Before etanercept or adalimumab was administered, CEUS resulted in being positive in 22 (88%) out of 25 subjects (Table 2).

At the end of the study, a complete remission of arthritis, defined as the absence of clinical and CEUS findings of joint involvement (see Section 2), was observed in 8 (32%) out of 25 patients (*P* < 0.004).

In 19 (86.3%) out of 22 patients in whom targeted joints (Table 1) were initially positive at CEUS examination, a decrease in the grade of contrast enhancement was observed (*P* < 0.0001) after 1 year of treatment (Table 2).

Among these 19 patients, with the exception of one subject (patient number 1), the reduction of contrast enhancement was in agreement with a decrease in the number of tender and swollen joints, as well as with regression of dactylitis and enthesitis (Table 2).

**Figure 1:**
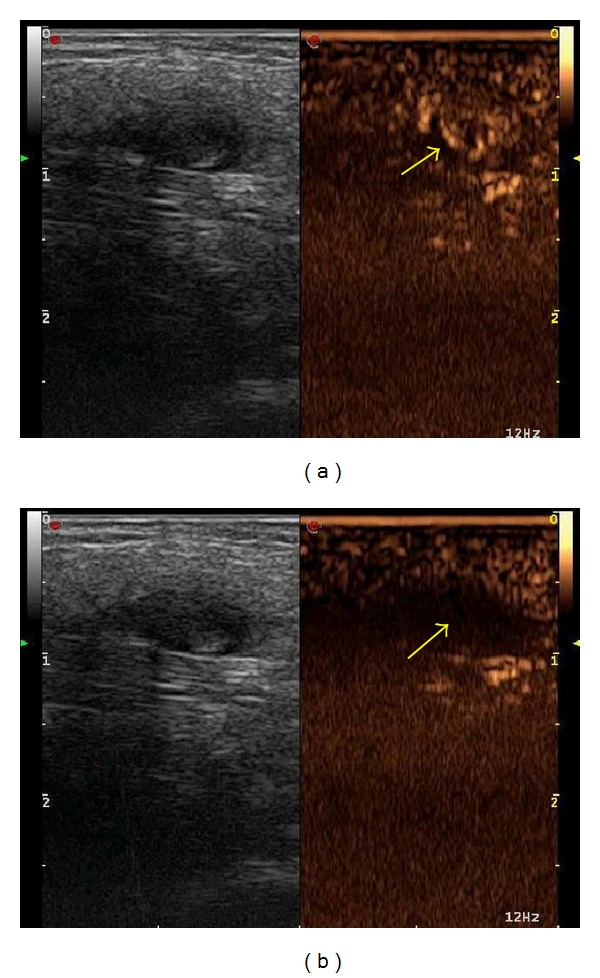
CEUS image of the I IF joint of the left hand (patient Number 25) at the start (a) and at the 12th month (b) of anti-TNF therapy. A reduction in the grade of contrast enhancement from (a) to (b) is shown.

A typical finding of intra-articular decrease of contrast enhanced signal from 3 (at the start of the therapy) to 0 (at 1 year of therapy) is shown in Figures 1(a) and 1(b), respectively.

The clinical outcomes of the three patients (patient numbers 4, 14, and 24) in whom a lack of reduction of the grade of contrast enhancement was observed at the 12th month of therapy are shown in Table 2. In particular, patient number 4 experienced an increase in the number of tender joints with a complete regression of the swollen joints and dactylitis. In patient number 14, an increase in the number of tender joints was observed, while patient number 24 continued to be positive at CEUS examination even if his inflammatory arthritis proved to be in complete clinical remission.

As regards the severity of psoriasis, the majority of patients at the start of the study had mild psoriasis with only 3 subjects (patient numbers 6, 14, and 15) with moderate to severe psoriasis (Table 2). The median PASI of the cohort of the patients was 2.7 (range: 0.0–20.4) prior to the administration of anti-TNF drugs. A significant reduction of PASI was observed (median: 0.0; range 0–6.6; *P* < 0.0001) after 1 year of therapy. However it must be noted that the sensitivity of the PASI method is low for psoriasis with a very limited extension [14].

**Figure 2:**
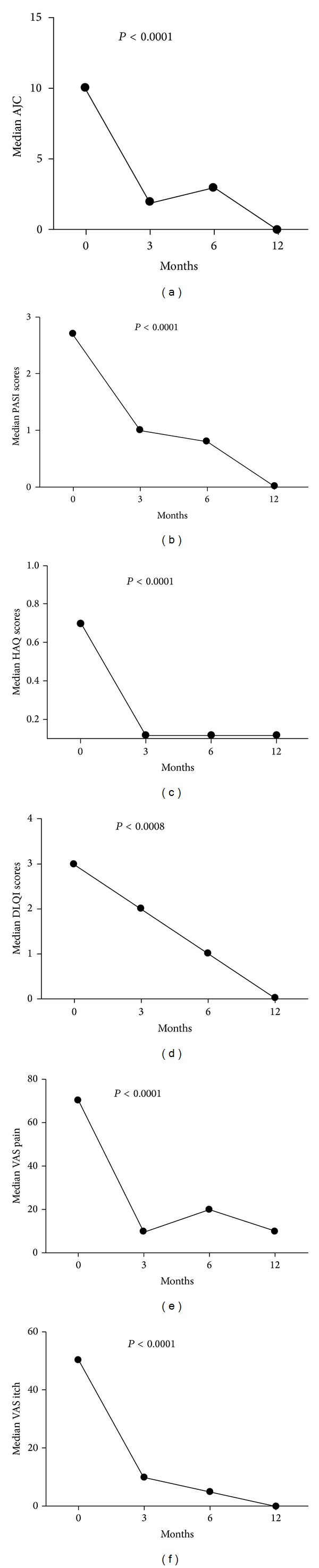
Median reductions from baseline over time in anti-TNF treated patients of (a) active joint count (AJC); (b) psoriasis area severity index (PASI); (c) health assessment quality (HAQ); (d) dermatology life quality index (DLQI); (e) Visual Analogic Scales (VAS) pain; and (f) visual analogic scales (VAS) itch.

The behaviours of the different parameters during the course of anti-TNF therapies are depicted in Figures 2(a)–2(f). Specifically, AJC, PASI, HAQ, DLQI, VAS pain, and VAS itch scores all showed a significant reduction.

No significant differences were observed for all the clinical variables considered in the present study, when the patients were grouped on the basis of the anti-TNF drugs administered (etanercept or adalimumab).

As regards ESR (range normal values: 2–25) and CRP (range normal value: 0.0–08 mg/dL) used as inflammatory markers at the beginning of the study, they were increased, respectively, in 9 (36%) and 8 (32%) out of 25 subjects. In particular, considering all the patients, the median ESR value was 19 (range: 2–71) and CRP 0.5 (ranges 0.0–2.4 mg/dL). Only 1 patient (patient number 1) continued to present high CRP concentrations (2.4 mg/dL) at the 12th month of therapy.

## 4. Discussion

The awareness of the risk of developing inflammatory arthritis in the context of psoriatic disease has prompted many efforts to optimize its diagnosis and treatment.

In the present retrospective study, we primarily wanted to verify the proportion of patients with PsA in whom there were no clinical or CEUS signs of joint inflammation after 12 months of effective anti-TNF therapy.

In the patients analysed herein, we focused mainly on the arthritic component because the majority of them presented only limited skin disease (see Section 3).

Response to therapy in PsA is often measured in terms of percentage of improvement versus baseline, but only a few data are available concerning the amount of patients reaching a remission of arthritis intended as a complete absence of signs of joint inflammation [15–19].

Our data show that after 1 year of continuous therapy with etanercept or adalimumab, a complete remission of joint inflammation, using the criteria expressed above (see Section 2), is obtained in 8 (32%) out of the 25 patients studied. This proportion was also more consistent, increasing from 8 to 11 patients (44%), if clinical criteria only were considered to define remission regardless of CEUS results.

At present, no remission criteria have been standardized for PsA and data reported so far indicate a frequency of clinical remission ranging from 0 [19] to 58% [20] with the highest rate observed in patients treated with anti-TNF drugs [18].

This wide range of the rate of remission reported is due to several reasons such as (i) type of patients studied, (ii) severity and duration of arthritis, (iii) therapies administered, (iv) presence of comorbidities, (v) type of studies (retrospective, prospective, controlled, or uncontrolled), (vi) settings in which the studies were conducted (i.e., dermatology or rheumatologic settings), and (vii) different criteria used to define remission.

Regarding the behaviour of the clinic parameters used in the present analysis (AJC, PASI, HAQ, DLQI, VAS pain, and VAS itching) a significant reduction was already observed as from the 3rd month of therapy that continued during the course of treatment up to the 12th month.

In our group of patients, no difference was detected between subjects taking etanercept or adalimumab with regard to improvement of all the variables cited above.

Our findings concerning the absence of difference between etanercept and adalimumab in improving inflammatory arthritis (AJC, HAQ, and VAS pain) are in keeping with data showing an equal effectiveness of anti-TNF drugs on joint inflammation in PsA [21].

As regards the comparative effectiveness of etanercept and adalimumab on skin symptoms in psoriatic patients, previously published data indirectly indicate a superiority of the latter drug [22].

In the series of patients we studied, we were unable to find any difference between etanercept and adalimumab in improving variables related to skin disease (PASI and DLQI). This finding is probably due to the small number of patients we studied as well as the low initial PASI score presented by the majority of them.

In the present study we used CEUS imaging technique to grade synovial inflammation because, as previously reported [7, 8], it has proved to be a sensitive technique to highlight synovitis.

Synovitis represents an important feature of PsA, together with enthesitis. In fact, synovial inflammation plays a key role in driving the structural joint damages seen in PsA [23, 24]. The ability of CEUS to detect the hyperemic stage of actively inflamed synovium is of particular interest in PsA. In fact, as well as in other seronegative spondyloarthropathies, and differently from rheumatoid arthritis, hypervascularity is an early feature of synovial inflammation also in PsA [24].

Our data show that 12 (54.5%) subjects out of 22 positive subjects at CEUS at the start of the study continued to present some grade of contrast enhancement at the level of targeted joints after 1 year of anti-TNF treatment. The persistence of positivity to CEUS signal, also in front of a reduction in its intensity as we found in 9 subjects studied (see Section 3), could have some important implications. In fact, it has been reported that anti-TNF drugs are able to slow or halt radiological disease progression [25–27]. However, the gold standard of a given therapy in a disease such as PsA is to switch off the inflammatory process at the level of joints. In fact, the persistence of an inflammatory status, also at low level, can only retard but not stop irreversible joint damages.

To conclude, data reported herein show that a complete remission of joint inflammation, defined by stringent criteria such as those used, could be obtained in a significant proportion of PsA patients after 1 year of continuous anti-TNF therapy.

However, the majority of patients examined (17/25), at the end of the study, still presented clinical and/or CEUS findings suggestive of a joint inflammatory status.

A followup longer than that reported herein with an increased number of treated patients is needed to verify the evolution of joint inflammation using the criteria we used in the present report.

## Figures and Tables

**Table 1:** Targeted joints examined by means of CEUS before and at 12 months of anti-TNF therapy.

Patient number	Joint
1	MCF II finger, left hand
2	MTF I toe, right foot
3	Right ankle
4	Left wrist
5	IFP IV finger, left hand
6	Right wrist
7	MTF I toe, left foot
8	Left ankle
9	IFP III toe, right foot
10	MCF III finger, left hand
11	IFP III finger, right hand
12	Left wrist
13	MTF I toe, right foot
14	MCF III finger, right hand
15	MTF III toe, left foot
16	IFD II finger, right hand
17	IFP II finger, left hand
18	IFP II finger, left hand
19	MTF I toe, right foot
20	IFP V finger, right hand
21	Right wrist
22	IFP II finger, right hand
23	IFP III finger, left hand
24	IFP II finger, left hand
25	IF I finger, left hand

MCF: metacarpal phalangeal joint; MTF: metatarsal phalangeal joint; IFP: interphalangeal proximal joint; IFD: interphalangeal distal joint; interphalangeal joint.

**Table 2:** Demographic, clinical, and CEUS characteristics of the patients before and at 12 months of anti-TN therapy.

Patient number	Gender	Age	Duration Pso (yrs)	Duration PsA (yrs)	Therapy	Month 0						Month 12					
						TJC	SJC	Dactylitis	Enthesitis	CEUS	PASI	TJC	SJC	Dactylitis	Enthesitis	CEUS	PASI
1	F	37	22	2	Adalimumab	10	0	No	No	2	0.2	10	0	No	No	1	1.0
2	M	45	24	15	Etanercept	18	1	No	No	3	3.7	0	0	No	No	0	0.0
3	F	27	25	3	Etanercept	19	0	No	Yes	3	0.0	0	0	No	No	0	0.0
4	F	48	6	<1	Adalimumab	48	7	Yes	No	2	0.9	51	0	No	Yes	2	0.0
5	M	55	3	3	Adalimumab	4	2	Yes	No	3	2.8	1	0	No	No	1	0.0
6	F	47	27	1	Adalimumab	12	3	No	Yes	3	10	4	0	No	Yes	1	2.4
7	F	63	21	21	Adalimumab	11	2	No	Yes	3	2.0	0	0	No	No	1	1.6
8	F	64	24	3	Etanercept	3	0	No	Yes	3	3.1	0	0	No	No	2	0.0
9	F	54	40	7	Etanercept	3	2	No	No	2	2.9	4	1	No	No	0	1.9
10	F	66	32	<1	Etanercept	10	6	No	No	1	2.7	8	0	No	No	0	0.8
11	M	48	4	1.5	Etanercept	17	2	Yes	Yes	3	1.2	1	1	No	No	0	0.0
12	M	58	24	14	Etanercept	1	1	No	No	0	2.7	0	0	No	No	0	0.0
13	M	41	23	1	Etanercept	2	1	No	No	2	2.6	0	0	No	No	0	0.0
14	M	54	36	16	Adalimumab	8	0	No	No	1	9.6	10	0	No	No	1	6.6
15	M	43	28	22	Adalimumab	17	6	No	Yes	3	20.4	1	0	No	Yes	0	0.0
16	F	59	46	20	Etanercept	2	2	Yes	No	0	3.2	0	0	No	No	0	1.8
17	M	36	2	1.5	Adalimumab	11	4	No	No	2	1.2	0	0	No	No	0	0.0
18	M	44	31	31	Etanercept	4	4	No	No	2	2.8	0	0	No	No	1	2.0
19	F	54	4	2	Adalimumab	15	3	No	Yes	3	2.4	15	0	No	Yes	1	0.0
20	F	51	15	<1	Adalimumab	8	2	No	Yes	3	0.8	2	0	No	No	1	0.0
21	M	62	10	<1	Etanercept	3	0	No	No	2	2.6	1	1	No	No	0	2.1
22	M	65	5	3	Etanercept	3	2	Yes	No	2	5.2	1	1	No	No	1	2.1
23	M	69	19	<1	Etanercept	6	0	No	No	0	5.9	0	0	No	No	0	5.0
24	F	29	8	3	Etanercept	10	1	No	No	1	0.2	0	0	No	Yes	1	0.0
25	M	45	24	2	Etanercept	9	2	Yes	No	3	4.5	0	0	No	No	0	3.6

TJC: tender joint count; SJC: swollen joint count; CEUS: contrast-enhanced ultrasound; PASI: Psoriasis Area Severity Index.
